# Supplementary material for: Pyruvate Dehydrogenase Kinase 1 inhibition mediated oxidative phosphorylation enhancement in cartilage promotes osteoarthritis progression
Source: BMC Musculoskelet Disord. 2023 Jul 20;24:597. doi: 10.1186/s12891-023-06585-6 (PMC10357736; doi:10.1186/s12891-023-06585-6)
Supplement: Supplementary file 1 — Additional file 1. [file 12891_2023_6585_MOESM1_ESM.zip › MF.Gsea.1653627316242/GOMF_OXIDOREDUCTASE_ACTIVITY_ACTING_ON_THE_CH_CH_GROUP_OF_DONORS.html]

Details for gene set GOMF\_OXIDOREDUCTASE\_ACTIVITY\_ACTING\_ON\_THE\_CH\_CH\_GROUP\_OF\_DONORS[GSEA]

|  || Dataset | 55457\_collapsed\_to\_symbols.cls.cls #OA\_versus\_normal.cls.cls #OA\_versus\_normal\_repos |
| Phenotype | cls.cls#OA\_versus\_normal\_repos |
| Upregulated in class | OA |
| GeneSet | GOMF\_OXIDOREDUCTASE\_ACTIVITY\_ACTING\_ON\_THE\_CH\_CH\_GROUP\_OF\_DONORS |
| Enrichment Score (ES) | 0.33332166 |
| Normalized Enrichment Score (NES) | 1.3526055 |
| Nominal p-value | 0.06878307 |
| FDR q-value | 0.37562585 |
| FWER p-Value | 1.0 |
Table: GSEA Results Summary

  

Fig 1: Enrichment plot: GOMF\_OXIDOREDUCTASE\_ACTIVITY\_ACTING\_ON\_THE\_CH\_CH\_GROUP\_OF\_DONORS      
 Profile of the Running ES Score & Positions of GeneSet Members on the Rank Ordered List

  

| SYMBOL | TITLE | RANK IN GENE LIST | RANK METRIC SCORE | RUNNING ES | CORE ENRICHMENT || 1 | DUS4L | dihydrouridine synthase 4 like [Source:HGNC Symbol;Acc:HGNC:21517] | 862 | 0.552 | -0.0208 | Yes |
| 2 | BLVRA | biliverdin reductase A [Source:HGNC Symbol;Acc:HGNC:1062] | 942 | 0.529 | 0.0174 | Yes |
| 3 | TECR | "trans-2,3-enoyl-CoA reductase [Source:HGNC Symbol;Acc:HGNC:4551]" | 1047 | 0.508 | 0.0519 | Yes |
| 4 | BLVRB | biliverdin reductase B [Source:HGNC Symbol;Acc:HGNC:1063] | 1050 | 0.507 | 0.0943 | Yes |
| 5 | ACAD8 | acyl-CoA dehydrogenase family member 8 [Source:HGNC Symbol;Acc:HGNC:87] | 1319 | 0.455 | 0.1116 | Yes |
| 6 | MECR | mitochondrial trans-2-enoyl-CoA reductase [Source:HGNC Symbol;Acc:HGNC:19691] | 1353 | 0.450 | 0.1467 | Yes |
| 7 | ACOX1 | acyl-CoA oxidase 1 [Source:HGNC Symbol;Acc:HGNC:119] | 1401 | 0.443 | 0.1802 | Yes |
| 8 | SDHB | succinate dehydrogenase complex iron sulfur subunit B [Source:HGNC Symbol;Acc:HGNC:10681] | 1818 | 0.382 | 0.1798 | Yes |
| 9 | SRD5A1 | steroid 5 alpha-reductase 1 [Source:HGNC Symbol;Acc:HGNC:11284] | 1973 | 0.364 | 0.1983 | Yes |
| 10 | PECR | peroxisomal trans-2-enoyl-CoA reductase [Source:HGNC Symbol;Acc:HGNC:18281] | 1985 | 0.363 | 0.2279 | Yes |
| 11 | AKR1C3 | aldo-keto reductase family 1 member C3 [Source:HGNC Symbol;Acc:HGNC:386] | 1996 | 0.361 | 0.2574 | Yes |
| 12 | SDHA | succinate dehydrogenase complex flavoprotein subunit A [Source:HGNC Symbol;Acc:HGNC:10680] | 2231 | 0.335 | 0.2673 | Yes |
| 13 | CPOX | coproporphyrinogen oxidase [Source:HGNC Symbol;Acc:HGNC:2321] | 3110 | 0.239 | 0.2190 | Yes |
| 14 | DHCR24 | 24-dehydrocholesterol reductase [Source:HGNC Symbol;Acc:HGNC:2859] | 3126 | 0.237 | 0.2377 | Yes |
| 15 | SDHC | succinate dehydrogenase complex subunit C [Source:HGNC Symbol;Acc:HGNC:10682] | 3145 | 0.235 | 0.2560 | Yes |
| 16 | CRAT | carnitine O-acetyltransferase [Source:HGNC Symbol;Acc:HGNC:2342] | 3159 | 0.234 | 0.2747 | Yes |
| 17 | ACOX2 | acyl-CoA oxidase 2 [Source:HGNC Symbol;Acc:HGNC:120] | 3176 | 0.233 | 0.2930 | Yes |
| 18 | DUS2 | dihydrouridine synthase 2 [Source:HGNC Symbol;Acc:HGNC:26014] | 3182 | 0.233 | 0.3121 | Yes |
| 19 | DECR1 | "2,4-dienoyl-CoA reductase 1 [Source:HGNC Symbol;Acc:HGNC:2753]" | 3272 | 0.224 | 0.3239 | Yes |
| 20 | ACADSB | acyl-CoA dehydrogenase short/branched chain [Source:HGNC Symbol;Acc:HGNC:91] | 3382 | 0.213 | 0.3333 | Yes |
| 21 | COX15 | cytochrome c oxidase assembly homolog COX15 [Source:HGNC Symbol;Acc:HGNC:2263] | 3727 | 0.183 | 0.3219 | No |
| 22 | DPYD | dihydropyrimidine dehydrogenase [Source:HGNC Symbol;Acc:HGNC:3012] | 3830 | 0.173 | 0.3285 | No |
| 23 | PPOX | protoporphyrinogen oxidase [Source:HGNC Symbol;Acc:HGNC:9280] | 4225 | 0.135 | 0.3092 | No |
| 24 | BDH2 | 3-hydroxybutyrate dehydrogenase 2 [Source:HGNC Symbol;Acc:HGNC:32389] | 4247 | 0.133 | 0.3187 | No |
| 25 | TBXAS1 | thromboxane A synthase 1 [Source:HGNC Symbol;Acc:HGNC:11609] | 4338 | 0.125 | 0.3222 | No |
| 26 | TM7SF2 | transmembrane 7 superfamily member 2 [Source:HGNC Symbol;Acc:HGNC:11863] | 4410 | 0.120 | 0.3267 | No |
| 27 | IVD | isovaleryl-CoA dehydrogenase [Source:HGNC Symbol;Acc:HGNC:6186] | 4950 | 0.076 | 0.2912 | No |
| 28 | ACADM | acyl-CoA dehydrogenase medium chain [Source:HGNC Symbol;Acc:HGNC:89] | 4983 | 0.074 | 0.2949 | No |
| 29 | ACAA1 | acetyl-CoA acyltransferase 1 [Source:HGNC Symbol;Acc:HGNC:82] | 5175 | 0.058 | 0.2849 | No |
| 30 | DHODH | dihydroorotate dehydrogenase (quinone) [Source:HGNC Symbol;Acc:HGNC:2867] | 5284 | 0.049 | 0.2806 | No |
| 31 | RETSAT | retinol saturase [Source:HGNC Symbol;Acc:HGNC:25991] | 5356 | 0.043 | 0.2787 | No |
| 32 | AKR1D1 | aldo-keto reductase family 1 member D1 [Source:HGNC Symbol;Acc:HGNC:388] | 5836 | 0.006 | 0.2420 | No |
| 33 | PTGES2 | prostaglandin E synthase 2 [Source:HGNC Symbol;Acc:HGNC:17822] | 6269 | -0.025 | 0.2105 | No |
| 34 | ACADVL | acyl-CoA dehydrogenase very long chain [Source:HGNC Symbol;Acc:HGNC:92] | 6394 | -0.034 | 0.2037 | No |
| 35 | ACADL | acyl-CoA dehydrogenase long chain [Source:HGNC Symbol;Acc:HGNC:88] | 6427 | -0.037 | 0.2043 | No |
| 36 | ACOX3 | "acyl-CoA oxidase 3, pristanoyl [Source:HGNC Symbol;Acc:HGNC:121]" | 6747 | -0.059 | 0.1845 | No |
| 37 | SRD5A3 | steroid 5 alpha-reductase 3 [Source:HGNC Symbol;Acc:HGNC:25812] | 7325 | -0.104 | 0.1483 | No |
| 38 | RSAD1 | radical S-adenosyl methionine domain containing 1 [Source:HGNC Symbol;Acc:HGNC:25634] | 7355 | -0.106 | 0.1550 | No |
| 39 | DECR2 | "2,4-dienoyl-CoA reductase 2 [Source:HGNC Symbol;Acc:HGNC:2754]" | 7476 | -0.116 | 0.1554 | No |
| 40 | GCDH | glutaryl-CoA dehydrogenase [Source:HGNC Symbol;Acc:HGNC:4189] | 8180 | -0.170 | 0.1149 | No |
| 41 | AKR1C1 | aldo-keto reductase family 1 member C1 [Source:HGNC Symbol;Acc:HGNC:384] | 8198 | -0.171 | 0.1279 | No |
| 42 | ACOXL | acyl-CoA oxidase like [Source:HGNC Symbol;Acc:HGNC:25621] | 8377 | -0.184 | 0.1295 | No |
| 43 | DHCR7 | 7-dehydrocholesterol reductase [Source:HGNC Symbol;Acc:HGNC:2860] | 8626 | -0.203 | 0.1271 | No |
| 44 | LBR | lamin B receptor [Source:HGNC Symbol;Acc:HGNC:6518] | 8834 | -0.220 | 0.1295 | No |
| 45 | ACADS | acyl-CoA dehydrogenase short chain [Source:HGNC Symbol;Acc:HGNC:90] | 9105 | -0.240 | 0.1286 | No |
| 46 | ACAD10 | acyl-CoA dehydrogenase family member 10 [Source:HGNC Symbol;Acc:HGNC:21597] | 9909 | -0.313 | 0.0923 | No |
| 47 | SRD5A2 | steroid 5 alpha-reductase 2 [Source:HGNC Symbol;Acc:HGNC:11285] | 10149 | -0.336 | 0.1019 | No |
| 48 | FASN | fatty acid synthase [Source:HGNC Symbol;Acc:HGNC:3594] | 12194 | -0.643 | -0.0033 | No |
| 49 | DUS1L | dihydrouridine synthase 1 like [Source:HGNC Symbol;Acc:HGNC:30086] | 12355 | -0.693 | 0.0424 | No |
Table: GSEA details [plain text format]

  

Fig 2: GOMF\_OXIDOREDUCTASE\_ACTIVITY\_ACTING\_ON\_THE\_CH\_CH\_GROUP\_OF\_DONORS      
 Blue-Pink O' Gram in the Space of the Analyzed GeneSet

  

Fig 3: GOMF\_OXIDOREDUCTASE\_ACTIVITY\_ACTING\_ON\_THE\_CH\_CH\_GROUP\_OF\_DONORS: Random ES distribution      
 Gene set null distribution of ES for **GOMF\_OXIDOREDUCTASE\_ACTIVITY\_ACTING\_ON\_THE\_CH\_CH\_GROUP\_OF\_DONORS**
